# The War Funds—Not by Public Subscription

**Published:** 1900-03-31

**Authors:** Henry Burdett


### Editor's Letter-Box.

[Our Correspondents are reminded that prolixity is a great bar to publication, and that brevity of style and conciseness of statement greatly facilitate early insertion.]

### THE WAR FUNDS—NOT BY PUBLIC SUBSCRIPTION.

Sir Henry Burdett writes: Money is now so readily obtained for any object connected however indirectly with the war that we are threatened by certain serious evils in consequence. Before these evils spread it seems desirable to insist that the State has certain distinct duties which cannot be superseded by charitable effort nor evaded by inertness on the part of the Government. It is the practice in this country, and, on the whole, an excellent one, for public subscriptions to provide the means whereby much good is done in days of emergency or calamity, or both. Still the public subscriptions raised in moments of excitement may become a danger if they are permitted to be asked without protest for objects which affect the honour and concern the character of the nation.

There are two instances which concern this miscarriage of procedure by public subscriptions. First, the widespread efforts made to provide convalescent accommodation for temporarily disabled soldiers and sailors on their return from the war. I am informed that upwards of 6,000 beds have been provided, most of which are and must remain unoccupied because the private soldier when discharged from hospital much prefers to go to his own home and friends. The letters I have received prove that the good people who have taken so much pains to provide these beds cannot yet realise Tommy's feelings in the matter, and are inclined to think that the War Office or the authorities at the military hospitals are purposely keeping soldiers out of the convalescent homes. The fact is that if the good people in question, instead of running away with the idea—a generous and pleasant idea I grant in the abstract—had first made inquiries and ascertained the facts, they would no doubt have decided to devote their energies to some other more practical field of work and would so have saved themselves much disappointment. Unfortunately some whose kindness of heart is universally recognised lack the judgment of leaders in philanthropic enterprises, and so lend their names to ill-considered proposals hurriedly formulated and frequently impracticable, as a little inquiry would convince the most sceptical.

The second instance relates to an enterprise equally well intentioned, equally impracticable, and it may even prove dangerous to the well being of the soldiers who are permanently disabled through war. What is the use of starting homes for soldiers and sailors of this class, to be maintained by charity, when the permanent provision of adequate means of support for all disabled soldiers and sailors is undoubtedly the business of the nation? There is a certain magnificence, no doubt, about the conception of a home to contain 250 disabled soldiers and sailors, but in practice such provision must prove but a drop in the ocean of suffering humanity from the army, whilst it will tend to do harm by diverting public attention from the plain duty of the Government, which may thus be encouraged to once more neglect the disabled warrior, as all Governments in this country have heretofore neglected him, after the close of each war. I see no objection to the provision of a site and the erection thereon of buildings for the reception of such disabled soldiers and sailors as may be willing to reside permanently in such a home; but it will be found, I believe, in practice that the permanently disabled soldier will share the feelings of his more fortunate comrades who are temporarily disabled, by manifesting a desire to go home, or at least to reside near the home of his family and friends. In any case the support of the permanently disabled soldier and sailor must devolve upon the Government, and I do most earnestly protest against the attempt now being made to raise £100,000 from the charitable for the purpose of providing for 250 men, because the principle which underlies this appeal is evil and not good, and ought, therefore, to be discouraged to the utmost by all who have the true interests of our disabled warriors really at heart. I desire not to be misunderstood. I fully admit the kindness of the thought which underlies the proposal, but I cannot fail to recognise the danger due to its utter inadequacy, the wrong lines upon which it is planned, and the undoubted abuse of attempting any such provision by public subscription. These objections have caused several eminent people to refrain from formulating plans which have been carefully thought out for the maintenance by private charity of the permanently disabled in the war. The idea now put forward is not new or original, therefore, and the objections to it are so generally admitted that I hope the appeal for the £100,000 for maintenance on reconsideration will be withdrawn.

No doubt all will agree that an adequate provision should be made for all soldiers and sailors who are permanently disabled in the service of their country. The nation can do no less than this. Probably the best way to secure it would be to actively promote a movement amongst County Councils and municipalities in favour of powers being conferred upon them to undertake to provide for the permanent support of disabled soldiers and sailors being natives of the districts which are within their jurisdiction. A privilege like this conferred upon local authorities would be welcomed, for it would embody the spirit which animates all classes of the people at the moment, and should render great service to the Government by making the army and the navy popular with all classes of the people in every part of the United Kingdom. Thus distributed, the charge upon each locality would be relatively inappreciable, and every part of the country could then arrange to bear their disabled heroes constantly in mind, and so continuously keep alive the military spirit and the duty which the ordinary citizen owes to those of his fellows who bear arms in the national defence.

Finally, I would say one word on behalf of our great home charities, which are in danger of being overlooked or pushed aside to-day by the innumerable war and other funds. Anyone who will pause to think for a moment will recognise that no one has the right to give to the war funds the money they usually contribute to the hospitals or to some other special charity in which they may be interested, and to leave the home charities with nothing. The usual contribution to the home charities should be regarded as sacred, and whatever is given for the purposes of the war should be given in addition to, and not in substitution of, the ordinary contribution to the local hospital or kindred charity. The London hospitals require each year about £150,000 in donations, but so far this year, I am informed, these institutions are badly in want of donations, which have almost ceased of late, a fact I commend to the charitable public in the hope that many people will lose no time in forwarding their usual contributions to the hospitals.

